# The *CD14 *functional gene polymorphism -260 C>T is not involved in either the susceptibility to *Chlamydia trachomatis *infection or the development of tubal pathology

**DOI:** 10.1186/1471-2334-5-114

**Published:** 2005-12-20

**Authors:** Sander Ouburg, Joke Spaargaren, Janneke E den Hartog, Jolande A Land, Johan SA Fennema, Jolein Pleijster, A Salvador Peña, Servaas A Morré, the ICTI consortium

**Affiliations:** 1 Laboratory of Immunogenetics, Section Immunogenetics of Infectious Diseases, Department of Pathology, VU University Medical Centre, Amsterdam, The Netherlands; 2 Department of Gastroenterology, VU University Medical Centre, Amsterdam, The Netherlands; 3 Public Health Laboratory, Municipal Health Service in Amsterdam, The Netherlands; 4 Department of Obstetrics and Gynaecology, Research Institute Growth and Development (GROW), Academisch Ziekenhuis Maastricht, and Maastricht University, Maastricht, The Netherlands; 5 STD outpatient clinic, Municipal Health Service in Amsterdam, The Netherlands

## Abstract

**Background:**

The functional polymorphism -260 C>T in the LPS sensing TLR4 co-receptor *CD14 *gene enhances the transcriptional activity and results in a higher CD14 receptor density. Individuals carrying the T/T genotype also have significantly higher serum levels of soluble CD14. The T allele of this polymorphism has recently been linked to *Chlamydia pneumoniae *infection. We investigated the role of the *CD14 *-260 C>T polymorphism in the susceptibility to and severity (defined as subfertility and/or tubal pathology) of *C. trachomatis *infection in Dutch Caucasian women.

**Methods:**

The different *CD14 *-260 C>T genotypes were assessed by PCR-based RFLP analysis in three cohorts: 1) A cohort (n = 576) of women attending a STD clinic, 2) a cohort (n = 253) of women with subfertility, and 3) an ethnically matched control cohort (n = 170). The following variables were used in the analysis: In cohort 1 the CT-DNA status, CT IgG serology status, self-reported symptoms and in cohort 2, the CT IgG serology status and the tubal status at laparoscopy.

**Results:**

In the control cohort the CC, CT and TT genotype distribution was: 28.2%, 48.2%, and 23.5% respectively. No differences were found in the overall prevalence of *CD14 *-260 genotypes (28.1%, 50.7%, and 21.2%) in cohort 1 when compared to the control cohort. Also no differences were observed in women with or without CT-DNA, with or without serological CT responses, with or without symptoms, or in combinations of these three variables. In subfertile women with tubal pathology (cohort 2, n = 50) the genotype distribution was 28.0%, 48.0%, and 24.0% and in subfertile women without tubal pathology (n = 203), 27.6%, 49.3% and 23.2%. The genotype distribution was unchanged when CT IgG status was introduced in the analyses.

**Conclusion:**

The *CD14 *-260 C>T genotype distributions were identical in all three cohorts, showing that this polymorphism is not involved in the susceptibility to or severity of sequelae of *C. trachomatis *infection.

## Background

*Chlamydia *species are related to a broad clinical spectrum of human disease including *Chlamydia pneumoniae *in lung and cardiovascular disease, *C. psittaci *in pulmonary emphysema and psittacosis, and *C. trachomatis *in ocular and urogenital infections [1-3].

*C. trachomatis *is the most prevalent sexually transmitted disease in Europe and the USA. Due to the mostly asymptomatic course of infection, these women will most likely not be treated resulting in an enhanced risk for the development of late complications, which include pelvic inflammatory disease (PID), ectopic pregnancy and tubal infertility.

The female reproductive tract is a very complex system where many factors, including hormones, vaginal flora and immune mediators, combine to provide protection on the one hand, while on the other hand maintaining an environment suitable for conception [4]. Clear differences in the clinical course of infection have been described and are due to an interaction between environmental (e.g. co-infection), bacterial (e.g. virulence factors) and host factors (genetic differences between individuals). In previous studies no clear associations have been demonstrated between *C. trachomatis *serotype, *C. trachomatis *genotype, and the course of *C. trachomatis *infection[5,6], although differences in cytotoxicity for different serovars have been described[7] and an association between *C. trachomatis *serovar G and cervical squamous cell carcinoma has been suggested [8]. In addition, virulence gene expression studies, and genomic comparisons of strains, isolated from clearly symptomatic or asymptomatic infected persons, revealed no strong role for the CT bacterium in relation to the course of infection[9,10].

A limited number of studies have recently demonstrated the influence of host genetic factors on the susceptibility to and the severity of *C. trachomatis *infection. Host factors including HLA-DQ and interleukin 10 (IL-10) have been associated with *Chlamydia *infection[11].

The Toll Like Receptor (TLR) family is a group of pattern recognition receptors, which recognise several microbial products, including bacterial cell wall components and DNA[12]. Poltorak *et al. *associated TLR4 with lipopolysaccharide (LPS) recognition in mice[13]. Further studies in mice corroborated these data [14,15], while studies in human demonstrated associations between TLR4 mutations and LPS hyporesponsiveness[16]. We did not observe an association between the *TLR4 *Asp299Gly polymorphism in patients with tubal pathology although the study population was relatively small[17]. The lack of association can be explained by recent publications showing that heterozygous carriage of the *TLR4 *Asp299Gly mutation does not affect LPS responsiveness and that only the rare homozygous carriers are less responsive to LPS[18].

**Figure 1:**
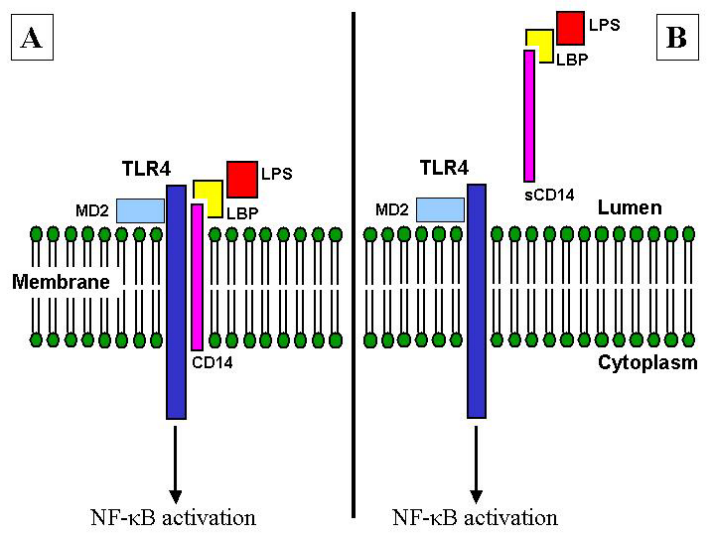
**CD14 localisation**. Panel A: Membrane-bound CD14 (mCD14) complexed with TLR4 and the LBP – LPS complex. Panel B: Soluble CD14 (sCD14). Abbreviations: TLR: Toll-Like Receptor; LBP: LPS Binding Protein; LPS: Lipopolysaccharide; NF-κB: Nuclear Factor κB.

CD14 acts as a co-receptor for TLR4 and confers responsiveness to LPS, a component of the cell wall of most Gram-negative bacteria. CD14 forms a complex with LPS and the LPS-binding protein (LBP) (figure 1) [19]. Combined with TLR4 this complex induces NF-κB associated immune responses including the release of a broad spectrum of cytokines that include tumour necrosis factor alpha (TNF-α), IL-1, IL-6, and IL-8 to initiate immune response[20].

The promotor region of the *CD14 *gene contains a single nucleotide polymorphism (SNP) at position -260. The -260 C>T genetic variation affects the binding of transcription factors[21] and has been associated with levels of sCD14 and inversely associated with serum IgE levels[20]. This SNP has been associated with myocardial infarction [22], Crohn's disease[23] and an increased susceptibility to develop chronic spondyloarthropathy in women[24].

Eng *et al. *demonstrated that carriers of the T allele of this promotor polymorphism have a higher expression of both mCD14 and sCD14 and that TNFα production is increased in the homozygous *CD14 *-260 T carriers when stimulated with either *C. pneumoniae *or *C. trachomatis *[25]. In a recent article, Rupp and colleagues described an association between the mutant allele and an increased susceptibility to chronic *C. pneumoniae *infection in coronary artery disease patients[26]. Since the *CD14 *-260 C>T is functional[25] and is associated with *C. pneumoniae *infection[26], one could hypothesize that in *Chlamydia trachomatis *infection this polymorphism could influence the susceptibility to and severity of this most prevalent sexually transmitted bacterium which is associated with female infertility.

Therefore, we investigated the role of the *CD14 *-260 C>T polymorphism in the susceptibility to and severity (defined as subfertility and/or tubal pathology) of *C. trachomatis *infection in Dutch Caucasian women. A cohort of women attending a STD clinic was used to assess the susceptibility to *C. trachomatis *infection, taking into account both *C. trachomatis *DNA and *C. trachomatis *IgG detection, symptoms and coinfections. A cohort of subfertile women with or without clinically well-defined tubal pathology was used to assess the role of CD14 in the severity of sequelae of *C. trachomatis *infection.

### Methods

#### Patient populations

##### STD cohort

Women of Dutch Caucasian (DC) origin (n = 576), under the age of 33 (range 14 to 33 years; median 22 years) and visiting the STD outpatient clinic in Amsterdam, The Netherlands, were included in this study (collection period: July 2001 – December 2004) (Table 1). All 576 women were consecutively included as the first part of a large prospective study. For every CT-DNA positive woman two consecutive CT-DNA negative controls were included in the study. The women were asked to sign an informed consent and to fill out a questionnaire, regarding their complaints at that moment, varying from increased discharge, having bloody discharge during and/or after coitus, recent abdominal pain (not gastrointestinal or menses related) and/or dysuria. A cervical swab was taken for the detection of *C. trachomatis *DNA (CT-DNA) by PCR (COBAS AMPLICOR; Hoffman – La Roche, Basel, Switzerland)[27].

**Table 1:** Patient characteristics in the STD and subfertility cohorts.

			**STD cohort**	**Subfertility cohort**
**n**			576	253
**CT DNA (LCx)**	+		184	
	-		392	
**CT IgG**	+		217	
	-		359	
**CT IgG (MIF)**	+ (> = 32)			41
	- (<32)			212
**Coinfections**	-		401	
	+		175	
		**C. albicans**	160	
		*N. gonorrhoea*	7	
		*T. vaginalis*	6	
		*H. simplex virus 1*	2	
		*H. simplex virus 2*	5	
**Symptoms**	-		335	
	+		221	
		Vulvovaginal discharge	157	
		Abdominal pain	81	
		Dysuria	58	
		Bleeding during/after coitus	25	
**Age**	Average		23.6 y	30 y
	Range		15 – 41 y	19 – 39 y
	Median		23 y	31 y
**Tubal Pathology**	+			50
	-			203

Abbreviations: CT: *C. trachomatis*; STD: sexually transmitted disease; TP: tubal pathology

Peripheral venous blood was collected for the analysis of IgG antibodies against *C. trachomatis *(CT) (Medac Diagnostika mbH, Hamburg, Germany). A titre of ≥ 1:50 was considered positive. Samples with grey zone values, e.g. cut off ± 10%, were repeated and considered positive when the result was positive or again within the grey zone. Infections with the microorganisms: *Candida albicans, Neisseria gonorrhoea, Trichomonas vaginalis, Herpes simplex virus 1/2*, may result in symptoms similar to CT infection. Infection status for these microorganisms was recorded. HSV 1/2 was detected according the methods described by Bruisten *et al*[28]. *N. gonorrhoea *was detected according methods described by Spaargaren *et al *[29]. *T. vaginalis *was cultured on Trichosel medium according standard procedures [30] and detection of *T. vaginalis *was according the methods described by van der Schee *et al *[31]. *C. albicans *was cultured on Chrom agar and detection of *C. albicans *was performed according standard procedures [30].

##### Subfertility cohort

The study was performed in 253 consecutive Dutch Caucasian women who visited the department of Obstetrics and Gynaecology of the Academisch Ziekenhuis Maastricht, The Netherlands, between December 1990 and November 2000 because of subfertility [32]. In these women a laparoscopy with tubal testing had been performed as part of their fertility work-up. Preoperatively blood was drawn from all patients for *Chlamydia *IgG antibody testing (CAT), and spare sera were cryopreserved.

Two independent investigators, who were unaware of the CAT results, scored the laparoscopy reports to assess the grade of tubal pathology. Tubal pathology was defined as extensive peri-adnexal adhesions and/or distal occlusion of at least one tube at laparoscopy [33]. Subfertile women who had no peri-adnexal adhesions and had patent tubes at laparoscopy served as negative controls. Based on these criteria, 50 women had tubal pathology and 203 women served as controls.

IgG antibodies to *C. trachomatis *were detected with a species-specific MIF test (AniLabSystems, Finland), as described previously [32], with comparable sensitivity and specificity as compared to the IgG ELISA from Medac used for the STD cohort [34]. A positive *C. trachomatis *IgG MIF test was defined as a titre ≥1:32. Findings at laparoscopy were correlated with the MIF test results. Based on the MIF test, 41 women were found to be CT IgG positive, while 212 were CT IgG negative. Of the CT IgG positive women 28 (68.8%) had tubal pathology, while 22 women (10.4%) of the CT IgG negative women had tubal pathology.

##### Healthy controls

A healthy Dutch Caucasian control group (n = 170) was included to assess the general frequency of the *CD14 *-260 genotypes in the Dutch Caucasian population.

#### Immunogenetic analyses

DNA Extraction

##### STD cohort

Eukaryotic DNA from PBMC was isolated using the isopropanol isolation method. In short: 100 μl PBMC in PBS were added to 600 μl L6 (Nuclisens Lysisbuffer, Organon Teknika, Boxtel, The Netherlands) and 1 μl glycogen (Roche Molecular Diagnostics, Almere, The Netherlands). The samples were incubated for 30 minutes at 65°C and left to cool at RT. An equal volume of cold (-20°C) isopropanol was added to the samples. The samples were then centrifuged (20 min at 20.000 G). The supernatant was discarded and the pellets were washed twice in 75% EtOH. The pellets were dissolved in T10 overnight (O/N) at 4°C and then stored at -20°C until further analysis.

##### Subfertility cohort

Genomic DNA was extracted out of the cryopreserved sera using High Pure PCR Template Preparation Kit (HPPTP kit) according to the manufacturer's instructions (Roche Molecular Biochemicals, Mannheim, Germany).

##### Healthy controls

Blood was collected in EDTA-tubes and stored at room temperature until the genomic DNA was extracted from peripheral blood leukocytes (PBMC) according to an in-house DNAzol (Invitrogen, The Netherlands) isolation procedure.

#### *CD14 *-260 C>T gene polymorphism

The C>T substitution in the proximal *CD14 *promoter GC box at position -260 from the translation start site (NCBI SNP CLUSTER ID:rs2569190) results in a *Hae*III restriction site. We developed a PCR assay using the primers, 5' TCA CCT CCC CAC CTC TCT T 3' (sense) and 5' CCT GCA GAA TCC TTC CTG TT 3' (antisense) (Invitrogen Life Technologies, Breda, The Netherlands), flanking this restriction site. Amplification was performed using a thermal cycler Perkin-Elmer 9700 (Applied Biosystems, Forter City, CA, USA). The parameters were an initial denaturation at 95°C for 5 min, followed by 35 cycles: denaturation at 95°C for 30 s, annealing at 59°C for 30 s, and elongation at 72°C for 1 min. The final elongation was at 72°C for 7 min followed for a cooling to 4°C. The 107-bp fragments were digested overnight at 37°C with *Hae*III (Invitrogen, The Netherlands) resulting in fragments that either were cut in two fragments of 83-bp and 24-bp (allele C) or were not restricted (T allele). These fragments were analyzed by electrophoresis on 4% low melting agarose gels (Tebu-Bio, The Netherlands) stained with ethidium bromide.

### Statistical analyses

All groups were tested for Hardy-Weinberg equilibrium to check for Mendelian inheritance. Statistical analyses were performed using Instat Graphpad and SPSS version 11 (SPSS Inc., Chicago, IL, USA). Fisher exact and χ^2 ^tests were used to test for differences in CD14 allele/genotype/carrier frequencies between the (sub)groups and p-values < 0.05 were considered statistically significant.

## Results

All genotype distributions assessed were in Hardy-Weinberg Equilibrium. The *CD14 *-260 C>T SNP was assessed in the STD, subfertility and control cohorts.

### *CD14 *-260 in the susceptibility to *C. trachomatis *infection

**Figure 2:**
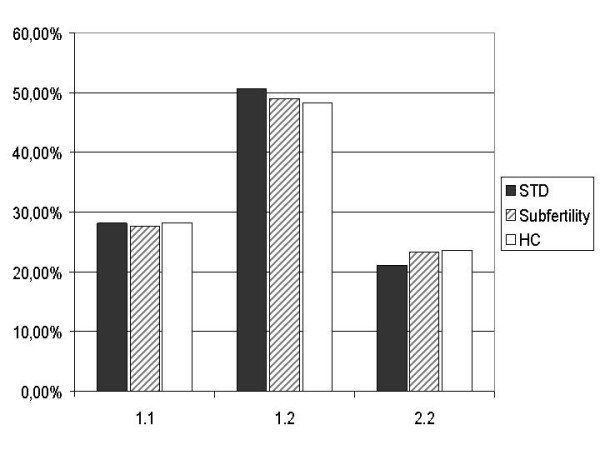
***CD14 *genotype distribution in the STD, subfertility and control cohorts**. Abbreviations: STD: sexually transmitted disease; HC: healthy controls

To determine the effects of *CD14 *-260 C>T on the susceptibility to *C. trachomatis *infection, the prevalence of *CD14 *-260 C>T genotypes were assessed in the STD cohort (table 2). The overall genotype distribution was 28.1% CC, 50.7% CT, 21.2% TT. This distribution was comparable to the healthy controls (figure 2). The distribution was 28.8% CC, 50.0% CT, 21.2% TT in CT DNA positive women, while in CT DNA negative women the distribution was 27.8% CC, 51.0% CT, 21.2% TT. In women with or without serological CT responses the distribution was 30.4% CC, 49.3% CT, 20.3% TT and 26.7% CC, 51.5% CT, 21.7% TT, respectively. No differences could be observed in women with or without symptoms. Coinfection with other microorganisms or combinations of these four variables (CT DNA, CT serology, symptoms and microorganisms) did not introduce statistically significant differences or trends in *CD14 *genotype distributions.

**Table 2:** *CD14 *genotype distribution in the Dutch Caucasian STD cohort.

					**CD14 -260 C>T**			
			**1.1 (CC)**		**1.2 (CT)**		**2.2 (TT)**	
		**Total**	**n**	**%**	**n**	**%**	**n**	**%**
	**Total**	217	66	30,4%	107	49,3%	44	20,3%
	
*CT IgG+*	**LCx+ (CT DNA+)**	135	38	28,1%	69	51,1%	28	20,7%
	**MO+**	42	12	28,6%	24	57,1%	6	14,3%
	**Symp**	56	14	25,0%	31	55,4%	11	19,6%
	**LAP+**	17	4	23,5%	10	58,8%	3	17,6%
	
	**LCx- (CT DNA-)**	82	28	34,1%	38	46,3%	16	19,5%
	**MO+**	29	12	41,4%	11	37,9%	6	20,7%
	**Symp**	43	16	37,2%	14	32,6%	13	30,2%
	**LAP+**	17	5	29,4%	8	47,1%	4	23,5%

	**Total**	359	96	26,7%	185	51,5%	78	21,7%
	
*CT IgG-*	**LCx+ (CT DNA+)**	49	15	30,6%	23	46,9%	11	22,4%
	**MO+**	16	3	18,8%	10	62,5%	3	18,8%
	**Symp**	19	5	26,3%	11	57,9%	3	15,8%
	**LAP+**	10	3	30,0%	6	60,0%	1	10,0%
	
	**LCx- (CT DNA-)**	310	81	26,1%	162	52,3%	67	21,6%
	**MO+**	88	20	22,7%	53	60,2%	15	17,0%
	**Symp**	103	26	25,2%	51	49,5%	26	25,2%
	**LAP+**	37	6	16,2%	20	54,1%	11	29,7%

*Healthy Controls*		170	48	28,2%	82	48,2%	40	23,5%

*C. trachomatis *IgG positive and negative patients, divided in CT DNA (LCx) positive and negative and subdivided in coinfection with other microorganisms (*N. gonorrhoea*, *T. vaginalis*, *C. albicans*, *H. simplex virus 1 & 2*), symptoms (vulvovaginal discharge, abdominal pain, dysuria, bleeding during/after coitus) and lower abdominal pain. Abbreviations: CT: *C. trachomatis*; MO+: microorganism positive; LAP+: lower abdominal pain positive; Symp: symptoms positive

### *CD14 *-260 in the severity of sequelae of *C. trachomatis *infection

The effect of *CD14 *-260 C>T on the severity of sequelae of *C. trachomatis *infection was assessed in a cohort of subfertile women with clinically well-defined tubal pathology. The overall genotype distribution in the cohort was 27.7% CC, 49.0% CT and 23.3% TT (figure 2). The genotype distribution in women with tubal pathology was similar to the distribution in women without tubal pathology (28.0% CC, 48.0% CT, 24.0% TT and 27.6% CC, 49.3% CT, 23.2% TT respectively) and to the distribution in the healthy controls (table 3). Introduction of CT IgG serology, with special attention to *C. trachomatis *positive women who did develop tubal pathology as compared to those who did not develop tubal pathology, did not alter the observed genotype distribution.

**Table 3:** CD14 genotype distribution in the Dutch Caucasian subfertility cohort.

				**CD14 -260 C>T**			
		**1.1 (CC)**		**1.2 (CT)**		**2.2 (TT)**	
	**Total**	**n**	**%**	**n**	**%**	**n**	**%**
*Total*	253	70	27,7%	124	49,0%	59	23,3%
**TP+**	50	14	28,0%	24	48,0%	12	24,0%
**TP-**	203	56	27,6%	100	49,3%	47	23,2%
**CT IgG+ TP+**	28	9	32,1%	15	53,6%	4	14,3%
**CT IgG+ TP-**	13	4	30,8%	6	46,2%	3	23,1%

**Healthy Controls**	170	48	28,2%	82	48,2%	40	23,5%

Distribution in the total cohort and subdivided in women with or without tubal pathology, and *C. trachomatis *IgG positive women with or without tubal pathology. *C. trachomatis *positivity defined as a titre ≥ 1:32 (MIF). Abbreviations: TP: tubal pathology; CT: *Chlamydia trachomatis*.

## Discussion

We did not find an association between the functional upregulating *CD14 *-260 C>T polymorphism and the susceptibility to or subsequent severity of sequelae of *C. trachomatis *infection, as assessed in the STD and subfertility populations (figure 2). However, these results do not exclude that a still unknown CD14 expression decreasing SNP may influence the course of *C. trachomatis *infection.

Recent studies have shown that *Chlamydia *LPS is capable of inducing an inflammatory response through CD14[35,36], although the potency to induce an inflammatory response was 100 – 1000 times less when compared to the responses induced by *S. minnesota*, *N. gonorrhoea *[35] and the enterobacteria *S. enterica *and *E. coli*[36]. Heine *et al. *demonstrated that the CD14 associated inflammatory response was TLR4 but not TLR2 mediated[36]. These results are corroborated by studies showing the role of the CD14-TLR4-MD2 complex in intracellular signalling by LPS[13,37] and studies showing the dependency on CD14 of phagocytosis of Gram negative bacteria [38].

The absence of an association between CD14 and susceptibility to *C. trachomatis *infection might be explained by the compartmentalisation of TLR4. The differential expression of TLR4 has been described in immortalised cell-lines derived from the female urogenital tract [39] and recently demonstrated in cells isolated from patients by Pioli[40] and Fazeli[41]. TLRs 1 – 6 were found to be expressed in the epithelia of the female urogenital tract. TLR2 and TLR4 were the only Toll like receptors with a clear differential expression. Low expression in the lower urogenital tract and high expression in the upper genital tract[40,41]. The expression remained similar in all subjects irrespective of age or status of the reproductive cycle[41]. It is hypothesized that through this expression pattern TLR4 modulates immunological tolerance in the lower genital tract and induces host defence against ascending infection in the upper genital tract[41]. In the upper genital tract, Fazeli and colleagues found TLR4 positive vacuole like structures that seemed to be secreted from endocervical glands[42]. A secretory form of TLR4 has been described in mice, where the soluble TLR4 appears to inhibit LPS mediated signals, while at the same time sTLR4 mRNA is upregulated by LPS[43]. This may represent a feedback mechanism to prevent excessive responses to LPS in the endocervix, which can be seen as a boundary between the lower and upper genital tract. Further evidence for the regulation of immune responses to LPS by TLR4 is provided by the study of Harju *et al.*, who demonstrated the intrauterine expression of TLR4 and endotoxin responsiveness in mice in the perinatal period [44]. mCD14 is expressed on human endometrial stromal cells but not on endometrial epithelial gland cells. The epithelial cells are dependent on sCD14 for LPS recognition [45]. Soluble CD14 is present in the cervical mucosa and may be present in the endometrium[46].

Combining the aforementioned studies with the knowledge that CD14 can signal through TLR4, it might be hypothesized that the absence of an association between the *CD14 *-260 SNP and the susceptibility to *C. trachomatis *infection might be due to the low expression or absence of TLR4 in the lower urogenital tract. In the upper genital tract, strict regulation of immune responses to LPS by TLR4 may inhibit CD14 signalling through TLR4 [43,44], thus limiting the influence of CD14 on the development of tubal pathology.

However, this hypothesis does not take into account the ability of CD14 to signal through TLR2[47], nor does it take into account that the study of Netea *et al. *which demonstrated that non-LPS components of *Chlamydia pneumoniae *can stimulate cytokine production through TLR2 dependent, CD14 independent pathways[48] and that a similar mechanism may exist and stimulate *C. trachomatis *induced cytokine production in urogenital infections.

Since TLR2 is involved in Chlamydia-induced TGF-beta, an anti-inflammatory cytokine with an important role in fibrosis, and thus very likely in post-infection tubal pathology, it might explain why CD14 polymorphisms may not severely impact the development of tubal pathology[49].

Darville *et al. *have demonstrated that TLR2 is an important mediator of innate immune responses in *C. trachomatis *infection in mice and plays an important role in early production of immune mediators and development of tubal pathology[50,51]. In a recent publication by Pitz *et al. *it was shown that *C. pneumoniae *is capable of activating endothelial cells by TLR2 as initial extracellular *C. pneumoniae *receptor, whereas NOD1 was shown to be a potent intracellular immune receptor for *C. pneumoniae *in endothelial cells. Further research may extend these results to *C. trachomatis *infections. Overall, the recognition of bacterial LPS involves a complex system of multiple receptors and a complex orchestration of protein-protein interactions [52].

## Conclusion

Our study showed that the functional up-regulating *CD14 *-260 C>T SNP did neither influence the susceptibility to nor the severity of late sequelae of *Chlamydia trachomatis *infection. However, this does not exclude a prominent role for CD14 in the course of an active *C. trachomatis *infection and not yet described CD14 expression decreasing SNPs may affect the course of *C. trachomatis *infection profoundly. Further studies on the immunogenetics of *C. trachomatis *infection will provide more insight in the clear differences in the clinical course that this microorganism induces in individuals and lead to potential vaccine candidates.

## Competing interests

The author(s) declare that they have no competing interests.

## Authors' contributions

SO: Data acquisition, data/statistical analyses, drafting the manuscript

JS: Sample collection, drafting the manuscript, critically revising for medical content

JAL and JEDH: Sample collection, critically revising for medical content

JSAF: Sample collection, critically revising for medical content

JP: Data acquisition, data analyses

ASP: Study design and conception, critically revising for immunogenetic content

SAM: Study design, conception and coordination, critically revising for immunogenetic content

*All authors contributed to writing of the final manuscript*.

All authors read and approved the final manuscript

## Pre-publication history

The pre-publication history for this paper can be accessed here:


